# Supplementary material for: Admixture into and within sub-Saharan Africa
Source: eLife. 2016 Jun 21;5:e15266. doi: 10.7554/eLife.15266 (PMC4915815; doi:10.7554/eLife.15266)
Supplement: Supplementary file 1. — DOI: http://dx.doi.org/10.7554/eLife.15266.034 [file elife-15266-supp1.zip › Busby_15-02-2016-RA-eLife-15266R1_Supplementary_File_1.pdf]

# Supplementary File 1 A note on ethnolinguistic groupings

The results of the population genetic analysis shows that population structure is largely the result of ethno-linguistic similarity, which itself is largely but not completely correlated with geographical proximity. These divisions are shown below and referred to in the text, together with the latest Ethnologue classification* of the languages spoken, where possible.

1. 1st major Niger-Congo speaking group from West Africa: Gambian and Malian ethnic groups
   - Niger-Congo, **Mande** {Mandinka, Malinke, Bambara}
   - Niger-Congo, Atlantic-Congo, Atlantic, **Northern, Senegambian, Fula-Wolof** {Fula, Wollof}
   - Niger-Congo, Atlantic-Congo, Atlantic, **Northern, Senegambian, Serer** {Serere, Serehule}
   - Niger-Congo, Atlantic-Congo, Atlantic, **Northern, Bak** {Jola}
2. 2nd major Niger-Congo speaking group from West Africa: Ghana/BF/Nigerian ethnic groups:
   - Niger-Congo, Atlantic-Congo, Atlantic, **Volta-Congo**, **Kwa**{Akan, Yoruba}
   - Niger-Congo, Atlantic-Congo, Atlantic, **Volta-Congo**, **North**, **Gur** {Mossi, Kasem, Namkam?}
3. A Central and Eastern African Niger-Congo / "Bantoid" speaking group, split into two subdivisions:
   (a) North Western
   - Niger-Congo, Atlantic-Congo, Atlantic, Volta-Congo, **Benue-Congo**, **exNarrow-Bantu** {Cameroon: Bantu, Semi-Bantu?}

   (b) Eastern
   - Niger-Congo, Atlantic-Congo, Atlantic, Volta-Congo, **Benue-Congo**, **Bantoid**, **Southern, Narrow-Bantu**, **Central**, **I** {Masaba-Luhya?}
   - Niger-Congo, Atlantic-Congo, Atlantic, Volta-Congo, **Benue-Congo**, **Bantoid**, **Southern, Narrow-Bantu**, **Central**, **E** {Mijikenda (Kenya)}
   - Niger-Congo, Atlantic-Congo, Atlantic, Volta-Congo, **Benue-Congo**, **Bantoid**, **Southern, Narrow-Bantu**, **Central**, **F-G** {Tanzania}
   - Niger-Congo, Atlantic-Congo, Atlantic, Volta-Congo, **Benue-Congo**, **Bantoid**, **Southern, Narrow-Bantu**, **Central**, **N** {Malawi (Chewa)}
4. Afroasiatic and Nilo-Saharan speakers from the Horn of Africa:
   - Afroasiatic, **Cushitic** {Afar, Somali, Oromo}
   - Afroasiatic, **Semitic** {Amhara, Tigrayan}

*information accessed on 28th July 2014 at https://www.ethnologue.com/

- Afroasiatic, **Omotic** {Ari, Wolayta}
- Nilo-Saharan, **Komuz** {Gumuz}
- Nilo-Saharan, **Eastern Sudanic**, **Nilotic** {Maasai}
- Nilo-Saharan, **Eastern Sudanic**, **Nilotic** {Anuak}
- Nilo-Saharan {Sudanese}

5. Khoesan and Bantu speaking groups from Southern Africa

   (a) Khoesan

   - Khoesan, Southern Africa, **Northern** {Ju/'hoansi, !Xun}
   - Khoesan, Southern Africa, **Central** {Nama}
   - Khoesan, Southern Africa, **Central**, **Tshu-Khwe**, **Northwest** {/Gui//Gana, Khwe}
   - Khoesan, Southern Africa, **Southern** {≠Khomani}
   - uncertain {Karretijie}

   (b) Southern Bantu speakers

   - Niger-Congo, Atlantic-Congo, Atlantic, Volta-Congo, **Benue-Congo**, **Bantoid**, **Southern, Narrow-Bantu**, **Central**, **R** {Herero}
   - Niger-Congo, Atlantic-Congo, Atlantic, Volta-Congo, **Benue-Congo**, **Bantoid**, **Southern, Narrow-Bantu**, **Central**, **S** {Amaxhosa, SEBantu}
