# Supplementary material for: Admixture into and within sub-Saharan Africa
Source: eLife. 2016 Jun 21;5:e15266. doi: 10.7554/eLife.15266 (PMC4915815; doi:10.7554/eLife.15266)
Supplement: Supplementary file 3. — DOI: http://dx.doi.org/10.7554/eLife.15266.036 [file elife-15266-supp3.zip › Busby_15-02-2016-RA-eLife-15266R1_Supplementary_File_3.pdf]

Supplementary File 3 Summary of inferred gene-flow from West to East and South Africa

In Figure 4-figure supplement 1 we show the changing composition of GLOBETROTTER's inferred sources of admixture. Figure 4-figure supplement 2 shows these same results with Niger-Congo surrogates only coloured, and with Malawi and Cameroon groups additionally emphasized. This analysis highlights several key insights:

1. **West and Central West Africa Niger-Congo.** Removing local surrogates from the West African Niger-Congo ancestry region does not substantially change the admixture source inference as admixture in groups from this region already involved Central West African donors. Sources of admixture in Central West African are replaced by West African Niger-Congo components in the non-local analysis. In both regions, admixture source components contain limited ancestry from East and South Africa regions. Interestingly, when we remove all West and Central West African surrogates, we observe similar major sources of admixture across groups from this region, which contains a significant amount of Nilo-Saharan / Afroasiatic ancestry (specifically Sudanese). We do not have Nilo-Saharan populations from Central and West Africa, although these exist. This observation may represent gene-flow between Nilo-Saharans populations across sub-Saharan Africa.

2. **East African Niger-Congo.** The major sources of admixture inferred in groups from this region tend to contain a majority of Southern African (Malawi) ancestry (Figure 4-figure supplement 1) whether or not local surrogates are removed from the analysis. When we remove East African Niger-Congo groups and Malawi from the analysis, other South African Niger-Congo speaking groups (SEBantu, Herero, Amaxhosa) are involved in the admixture source. Subsequent removal of these groups leads to an admixture source that is almost exclusively made up of groups from Cameroon, suggesting that Niger-Congo ancestry in East and Southern Africa is most closely related to populations from this part of Central West Africa. Intriguingly, the remainder of major admixture source is made up of a small amount of Khoesan ancestry. Whilst we do not have autochtonous groups from East Africa, this component likely represents ancestry from groups that were present in the area before the major West to East population migrations.

3. **East African Nilo-Saharan and Afroasiatic.** In the full analysis the major source of admixture tends to contain local (purple) groups (Figure 4-figure supplement 1). When we remove local surrogates from the analysis we see an increase in West and East African donors in the major sources of admixture.

4. **South African Niger-Congo.** We similarly observe mostly East African Niger-Congo ancestry in South African Niger-Congo speakers whether or not we remove non-local surrogates, although there is also a significant Malawi component in the SEBantu and Amaxhosa in the full analysis, which is replaced by East African Niger-Congo groups in the non-local analysis. As in East African Niger-Congo speakers, when we disallow any East or South African Niger-Congo surrogates, the major sources of admixture are almost exclusively from Cameroon donors.

5. **South African Khoesan.** Several Khoesan groups show evidence of specific admixture events involving Central West African donors, in the Khwe, /Gui//Gana, and Xun. When we remove Khoesan groups from being surrogates, interestingly we see that the major sources of admixture now tend to contain ancestry from Southern African Niger-Congo populations, which specifically are not Malawi-like (Figure 4-figure supplement 1). We also observe a small amount of West African (dark blue) ancestry in these admixture sources, and very little Central West African ancestry (and none from Cameroon). This suggests that the non-Khoesan ancestry in groups from this region is unlikely to be associated with the migrations that spread the Cameroon (Bantu) ancestry into East and South Africa. We also observe a small amount of East African Nilo-Saharan / Afroasiatic ancestry in the major sources of admixture in the non-local analysis.

A key insight from these analyses is that a large amount of the ancestry in East and Southern Africa Niger-Congo speaking groups comes from populations currently present in Central West Africa. The opposite is not true for Western and Central West African groups, who share DNA amongst themselves, but when local surrogates are removed, they instead choose mainly non-Niger-Congo speaking groups as admixture sources.
